# Managing abusive experiences: a qualitative study among older adults in Sweden

**DOI:** 10.1186/s12877-022-03143-y

**Published:** 2022-05-26

**Authors:** Johanna Simmons, Nicolina Wiklund, Mikael Ludvigsson

**Affiliations:** 1 Department of Acute Internal Medicine and Geriatrics in Linköping, and Department of Health, Medicine and Caring Sciences, Linköping University, Linköping, Sweden; 2 Department of Psychiatry in Linköping, and Department of Biomedical and Clinical Sciences, Linköping University, Linköping, Sweden

**Keywords:** Elder abuse, Polyvictimization, Coping, Help-seeking behaviour, Social support

## Abstract

**Background:**

Elder abuse is prevalent, and is associated with poor health outcomes. How an older adult is affected by abusive experiences is dependent on myriad factors, including aspects of the abuse itself, other life circumstances, coping strategies, and what kind of help the older adults receive to manage the experience. In this study, we sought to investigate how older adults themselves describe how they manage abusive experiences. An increased understanding of this could help to tailor society’s response to older adults suffering from abuse.

**Method:**

Participants (*n* = 30) were recruited from patients admitted to one acute geriatric and one acute internal medicine ward at a university hospital in Sweden. Patients over the age of 65 who reported experiences of elder abuse or who reported that they were still suffering from abuse that had occurred earlier in life were included. In-depth qualitative interviews were conducted, transcribed verbatim, and analyzed using qualitative content analysis.

**Results:**

The analysis resulted in five themes, three pertaining to strategies used to manage abusive experiences (self-reliant coping, restoring dignity in relation to others, and needing formal and informal help) and two pertaining to the disclosure process (inner resistance to disclosure, and external barriers and facilitators for disclosure).

**Conclusion:**

Older adults were found to use a combination of different strategies to manage abusive experiences. Some were self-reliant, but older adults often managed their experiences with the help of others. Health care professionals were generally in a position to facilitate disclosure, but some participants reported poor encounters with health care. The findings indicate a need to facilitate disclosure by, for example, training professionals on issues related to elder abuse and developing more easily navigated response systems that can respond to the complex needs of older adults trying to manage abusive experiences.

**Supplementary Information:**

The online version contains supplementary material available at 10.1186/s12877-022-03143-y.

## Background

In 2017 a global meta-analysis reported that one in six community dwelling older adults have been subjected to elder abuse [1]. The prevalence in institutional settings is more difficult to estimate, but is likely to be much higher [2]. In Sweden, the reported prevalence rate of elder abuse has been reported between three and 31% [3–5]. The considerable differences between studies are due to varying definitions and operationalizations used in different studies. This methodological problem has been repeatedly reported in studies on elder abuse internationally as well as, more specifically, in Swedish studies about intimate partner violence [6–8]. In this study we use the definition of elder abuse adapted by the World Health Organization in the Toronto Declaration stating that “Elder abuse is a single or repeated act, or lack of appropriate action, occurring within any relationship where there is an expectation of trust which causes harm or distress to an older person” [9]. It can occur both at the hand of intimate partners, adult children, other family members and professionals and can be of various forms: physical, emotional, sexual, financial or simply reflect intentional or unintentional neglect [9]. In the last years, it has also become increasingly clear that many victims of elder abuse are polyvictims, i.e., they are suffering from multiple co-occurring or sequential types of abuse by one or more perpetrators [10–12]. In addition, abuse in childhood has been found to be associated with increased odds of also reporting elder abuse [13–15]. Hence, considering polyvictimization and using a life-course perspective are important in research into elder abuse [16–20].

Although elder abuse has a profound effect on victims’ lives, little is known about which coping strategies older adults use to manage their experiences. Coping strategies can be classified in different ways. One important distinction is between disengagement and engagement coping [21]. Disengagement coping occurs when someone avoids the stressful events and emotions that follow from the stressor. Common strategies for disengaging include denial, avoidance, and substance abuse. By contrast, engagement coping strategies are directed toward reducing or eliminating the stressor or the emotional response. Engagement coping strategies can be problem-focused or rather emotionally focused, e.g., seeking emotional support from others [21]. Disengagement coping strategies may be helpful for the individual in the acute phase after abuse or a crisis [22]. However, in the long run, engagement coping strategies are generally considered adaptive and disengagement coping strategies maladaptive [21, 23, 24].

Seeking help can be considered a form of engagement coping. However, non-use or underuse of community services for elder abuse victims has been repeatedly reported [25]. In the U.S. National Elder Mistreatment, study only 15% of victims had sought help from authorities [26]. In Sweden, only 2% of older adults had ever been asked questions in health care about violent experiences, although in the same study 25% reported some form of life-course abuse [27]. At the same time, victims report needing more help than they currently receive and many report uncertainty about how to navigate between support services [12, 28]. Sweden does not have mandatory reporting of elder abuse or an authority specially devoted to protecting vulnerable older adults. Considering this, the health care system may play an important role in identifying and caring for older adults subjected to abuse. Unfortunately, many victims of elder abuse refrain from seeking help until the abuse is very serious or perceived as unbearable [12, 29, 30]. Common barriers for help-seeking are shame, self-blame, and fear of negative consequences, e.g., worsening of the abuse or being institutionalized [29]. Also, older adults often refrain from seeking help or participating in interventions when they are dependent on the perpetrator or if they perceive that help-seeking will have a negative impact on the family, often including the perpetrator of abuse [12, 26, 29, 31, 32]. It is also likely that older adults’ previous life experiences will affect their attitudes toward seeking and receiving help [33].

A number of qualitative studies concerning the lived experience of elder abuse and help-seeking have been conducted in recent years [31, 32, 34]. However, more research is needed to gain a comprehensive picture of how older adults manage abusive experiences. Such knowledge is essential to be able to improve the societal response for older adults suffering in the aftermath of abuse. For example, little research has been directed at what facilitates the disclosure of elder abuse [35]. Also, much of the research comes from the perspective of professionals rather than from the older adults themselves, and it is common to use victim services to recruit participants. The perspective of older adults who have never sought help may hence be overlooked. To make the societal response as relevant as possible, the voices of victims themselves need to be heard to a greater extent [29, 30, 33, 36] and studies using samples from different contexts need to be conducted, in order to broaden the understanding of how older adults manage abusive experiences.

The aim of this study was therefore to explore how older adults who report elder abuse, or that they are still suffering from abuse that occurred earlier in life, manage their experiences of abuse, including the process of disclosing abuse to formal or informal helpers. An analysis of the participants’ experiences of elder abuse is presented separately [20].

## Method

This study was part of the larger Responding to Elder Abuse in GERiAtric care (REAGERA) project conducted in Sweden [37]. The participants were recruited among in-patients aged ≥ 65 years on one acute geriatric and one acute internal medicine ward at a university hospital in Sweden. Patients were recruited consecutively, and were asked to fill out a screening instrument (REAGERA-S) for detecting elder abuse as well as experiences of abuse earlier in life [37]. Exclusion criteria were having insufficient cognitive, physical, or language capacity to be able to read and fill out the screening instrument, either independently or with the assistance of a care professional. The procedure for recruiting participants has been described in detail elsewhere [37]. In total, 306 participants were asked to participate, 191 answered the screening instrument, and 135 older adults agreed to participate in an interview to validate the results of the screening instrument. In cases where abusive experiences were revealed, an in-depth qualitative interview was conducted, and it is the data from these interviews that were analyzed for this study. All those reporting elder abuse or experiences of abuse earlier in life that they still suffered from were included (*n* = 30). Originally, the focus was intended to be only on managing experiences of elder abuse. However, as has been previously described, it was evident from the interviews that the experience of elder abuse was affected by previous life-experiences, including victimization [20]. Also, the level of suffering or need of help was not only related to when in time the abuse had occurred. Some older adults who reported abuse only in childhood or adulthood still suffered considerably from this, and in several cases they had never sought help. As we consider it important to improve the health care response and facilitate help-seeking for this group, we decided to also include informants who reported that they were still suffering from abuse earlier in life in this study. Such emergent design flexibility corresponds to general recommendations for qualitative research [38]. Older adults who only reported experiences earlier in life and no suffering were not included. Poly-victimization was common, among the 30 informants included in this study; ten only reported elder abuse, six only reported abuse earlier in life, and fourteen reported both abuse earlier in life and elder abuse. Most informants had lifetime experiences of emotional (*n* = 24, 80%) or physical (*n* = 17, 57%) abuse, but experiences of neglect (*n* = 13, 43%) as well as sexual (*n* = 12, 40%) or financial (*n* = 6, 20%) abuse was also common. The background characteristics of the informants are presented in Table 1.

**Table 1:** Background characteristics of included participants (*n* = 30)

		**n**	**%**
**Age**			
65–74 years		11	37.9
75–84 years		8	27.6
≥ 85 years		10	34.5
**Sex**			
Women, n (%)		20	66.7
Men		10	33.3
**Educational level**			
≤ 9 years		13	43.3
10–12 years		9	30.0
≥ 13 years		8	26.7
**Living arrangements**			
House or apartment		26	89.7
Assisted living		3	10.3
Living alone		18	62.1
Living with partner		11	37,9
**Need for help with ADL**			
No need		11	37.9
Help with instrumental ADL		10	34.5
Help with basic ADL		8	27.6
**Receive help with ADL from professionals**^a^			
No		16	55.2
Yes		13	44.8

ADL activities in daily living. Instrumental ADL = e.g., medications, groceries, and housework. Basic ADL = e.g., eating, dressing, and personal hygiene. Missing cases = 0–1

^a^professionals = staff at assisted living facility or home care staff.

Interviews were conducted in a private room at the hospital by one of the three authors. The interview revolved around how informants had coped with their experiences of abuse, their help-seeking experiences, and their perceptions of how the health care response to victims of abuse can be improved. The interview guide can be found as additional file 1. Interviews were recorded and transcribed verbatim. Non-verbal expressions such as sighs and laughter were also noted in the transcripts, and the interviews lasted between 12 and 96 min.

**Fig. 1:**
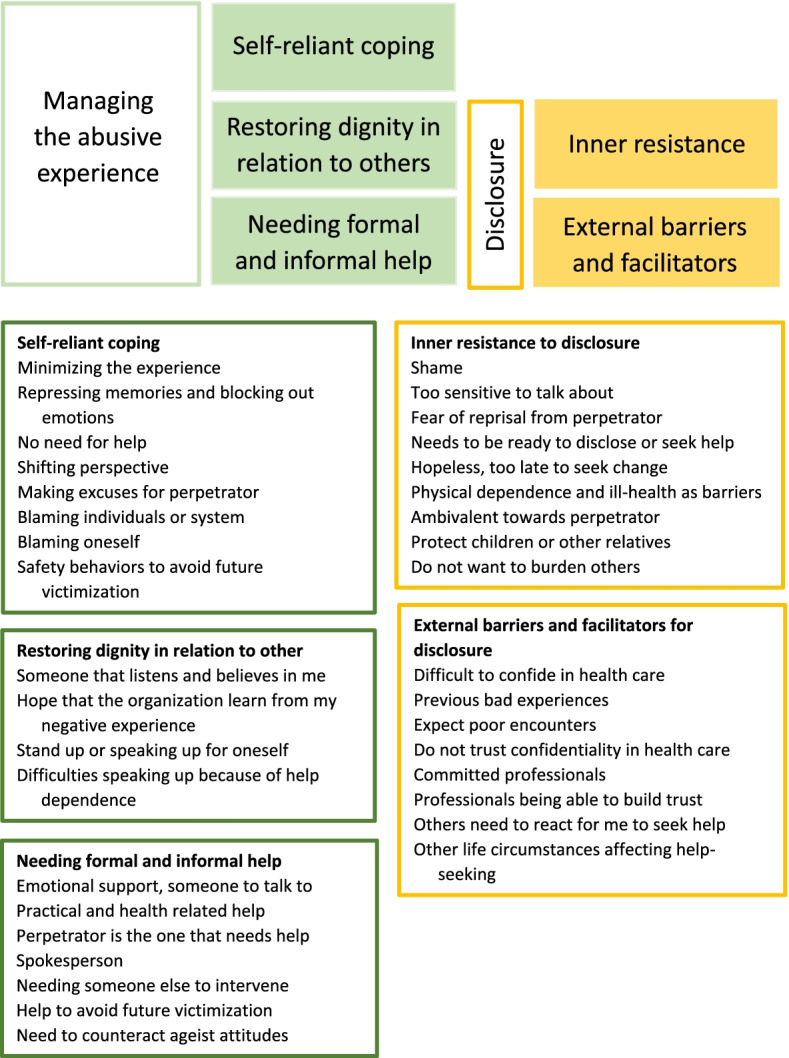
Summary of results; top part of the figure is an illustration of the themes and how they relate to each other while bottom part represents a simplified coding scheme, naming categories that make up each theme

The analysis was conducted according to qualitative content analysis as described by Graneheim and Lundman [39]. The first author (JS) coded all interviews and the last author (ML) coded six of the interviews to ensure similar interpretations of the text. The following steps were carried out: 1) Transcripts were read and re-read in order to grasp the overall sentiment of the interviews. 2) Transcripts were then divided into meaning units, which were subsequently condensed. 3) The underlying meaning was interpreted, and the condensed meaning units were abstracted into codes. 4) Codes were compared based on differences and similarities, and were grouped into categories which constitute the manifest content of the data. 5) The authors thereafter reflected on and discussed how the categories related to each other and the latent content of the interviews. This process resulted in the five themes which are presented together with the categories in Fig. 1. In the analysis, we searched for convergent patterns but also investigated divergence, i.e., we considered data that did not fit the dominant patterns. An example of the coding is presented in Table 2.

**Table 2:** Example of coding scheme. In this example the categories that constitute the theme “Restoring dignity in relation to others” is displayed. Each category is made up of several codes but only one example for each category is presented here. Likewise, each code is made up of several condensed meaning units, but only one meaning unit for each code is presented here

Meaning Unit (quote)	Condensed meaning unit	Code	Category	Theme
About the desired response from the health care system after telling about abuse: *“Absolutely believe the person that tells. That is the first thing. That is absolutely most important, and not be sceptical, and sort of “No, well… perhaps…” That is a very common attitude. Instead, one has to truly believe what is said, and then in all ways possible give support.”*	In health care: Absolutely believe the one that tells [about abuse]. That is the first thing. That is absolutely most important […] one has to truly believe what is said, and then in all ways possible give support	Most important is that the health care staff believes in me and give support	Someone that listens and believes in me	Restoring dignity in relation to others
About abuse in health care: *“When physicians have some sort of meeting and have lectures and so on, one could bring this up as an example”*	When physicians have some sort of meeting […] one could bring this up as an example	Use my experience to learn from	Hope that the Organization learn from my negative experience	
About abuse by a partner *“Do it one more time and I will bust you for it. Then he started laughing. You can laugh I said, but you won’t laugh if you do it, because I promise I will report you to the police”*	Do it one more time and I will bust you for it. […] you won’t laugh if you do it, because I promise I will report you to the police	Speaking up against the abuser	Stand up or speaking up for oneself	
About abuse at a nursing home: *Sometimes I get so mad and can not fall asleep when things have happened. Then I lay thinking about it and get angry. So I feel kind of powerless.”*	I get so mad and can’t sleep. Then I lay thinking about it and get angry. I feel kind of powerless	Feel powerless when abuse occurred at nursing home	Difficulties speaking up because of help dependence	

Field notes were taken during both the interview process and the coding. Field notes made immediately after the interviews contained memos about the most striking part of the interview, including how that related to preconceived ideas about elder abuse. We also made notes about the situation that could later be used to recall the specific interview, e.g., about the room used or some other specificities about the interview situation. In the beginning of the coding process, at step 1 (reading and re-reading the transcripts), new notes were made about what was found to be the most important content of the interview as well as about thoughts and questions that emerged when reading the transcript. Thereafter the more detailed coding took place in step two and three. In step four and five (when categories and themes were created) we returned to the notes taken during the process. This ensured reflexivity in the coding process and that we did not lose track of the overall content of the interviews while paying attention to details.

## Results

Five themes were identified, and are presented in detail below. Three of these relate to how older adults actually manage abusive experiences: 1) self-reliant coping strategies, 2) restoring dignity in relation to others, and 3) needing formal and informal help. The remaining two themes pertain to factors influencing disclosure of abusive experiences, a prerequisite for both restoring dignity in relation to others and help-seeking: 4) inner resistance to disclosure and 5) external barriers and facilitators for disclosure (Fig. 1).

### Self-reliant coping strategies

One recurrent strategy for managing the experiences of abuse was either to minimize the significance of the abusive experiences or to repress emotions and memories of it, i.e., a form of avoidance coping. Many respondents talked about putting the abusive experiences behind them and avoiding thinking about what had happened, or repressing their feelings. For some, this was an active decision, but for others it occurred more passively. One woman who had been subjected to emotional and controlling violence from a family member for most of her life said:

*“So many things have happened, so my feelings are kind of numbed.” (Woman, aged 85)*

For some, this seems to have been a successful strategy. However, for others it was instead a fruitless attempt to escape difficult feelings. Over time, different circumstances often triggered a reaction to or a recollection of the abuse, sometimes long after it had occurred.

Another coping strategy used by some respondents was similar to behavioral activation. By shifting the perspective and for example deciding to create a positive atmosphere for their children or to care for their grandchildren, the informant consciously decided to focus on something valued as positive instead of the abusive experience. One woman talked about financial abuse by home care personnel. Her niece wanted her to report it to the police, but she herself did not want to use her limited energy during her final years in life for such negative things:

*“Perhaps my niece said to me ‘Should we get it [the police report] going…?’ and then perhaps I said that I do not have the energy… one does not really have the energy for negative things.” (Woman, aged 73)*

One way to cope with the experience of abuse was by trying to understand why it had happened and attributing guilt. Some focused on the individual responsibility of the home care staff, and said that they trusted the permanent staff but not the temporary staff. Others blamed the health or social care recruitment process for letting in employees who were not suited for the job. Some older adults subjected to neglect in health care instead expressed an understanding of the abuser but blamed the system, i.e., a lack of time and resources for health and social care workers.

*”They [the home care staff] have certain time [limits], 5 minutes here and there […] Yes I understand, a lot of things to do maybe, many things that puts a pressure on them” (Woman, aged 84)*

Excuses for the abuser were also made by older adults suffering in other kinds of relationships, e.g., a woman suffering from abuse by her husband blamed it on alcohol.

Blaming oneself for the abuse was common, and some reflected on different ways they could change their own behaviors to avoid future victimization. For example, home care personnel had stolen jewelry from one older woman, and she blamed herself for leaving her jewelry box in plain sight. To protect herself from future victimization, she had made plans to start hiding her valuables and to start noting the names of the personnel that came to care for her.

*“I had thought beforehand that I would re-arrange some of the things at home. So that… so there is no temptation [for the home care staff]. […] And now when I called [for help due to my illness], I crawled up… I have barely any jewelry left at home, but I put away the jewelry box, so as not to leave it out.” (Woman, aged 73)*

### Restoring dignity in relation to others

Interactions with others, e.g., professionals, friends, and family, were important for the process of restoring one’s dignity. Signs of respect from others, e.g., being listened to and believed, as well as signs of self-respect, e.g., standing up for oneself to prevent abuse, was a recurrent theme in the interviews. The need to be taken seriously, to feel respected, and to feel validated was repeatedly emphasized by the informants. One woman subjected to childhood abuse as well as intimate partner violence during many years of adult life realized that it might not be in the health care providers’ power to change her life circumstances, but she returned to the importance of being believed. When asked for her advice to health care professionals, she responded:

*“Well, first of all, the very first thing, is that they believe what you say, that is the most important thing. And then, if they can do something to stop it [the abuse], they should do it. But anyway, they should listen, and they should believe what you say. And you should not get a lot of crap thrown back in your face. That is all I have to say.” (Woman, aged 72)*

Likewise, the participants conveyed that it was important for formal complaints to be taken seriously. A few informants had submitted police reports about financial abuse by health care professionals. Although they could rarely explain what had happened with their report or in some cases knew that the report had been dropped, it was important for them that it had been taken seriously by the police officer or the manager responsible for the accused health care professional. When abuse had occurred in health or social care, one expression for the desire to be taken seriously was a wish that the health care system should learn and take active measures so that abuse did not occur again. Some suggested that their stories should be brought up at internal staff training sessions, so that staff could learn from what had happened to them and prevent similar abusive events in the future. For some, it seemed to be a comforting thought that their negative experiences could help to prevent others being abused.

*”What I think is that the health care system should know about it […] When physicians have some sort of meeting and have lectures and so on, one could bring this up [my abusive experience] as an example” (Woman, aged 88)*

Another side of being restored in relation to others was standing up for oneself. Informants described how they had taken a stand and expressed that the abusers had no right to treat them in an abusive way. One woman talked about a man who used to live in the same nursing home as her, and who was rolling around in his wheelchair, yelling and threatening residents and staff: 

*“I gave him an earful and then… and then he was swept away from here.” (Woman, aged 81)*

Several informants found it more difficult to stand up for themselves and their rights when there was a dependence embedded in the relationship, e.g., in contact with health care professionals. Also, examples of standing up for oneself were mostly described by those respondents who had been abused for the first time as older adults and less so by polyvictims. By contrast, a few older adults described abusive experiences throughout life but that they had started to stand up for themselves at an older age, either by speaking up against the abuse or by threatening a police report.

### Needing help from formal and informal sources of support

Most informants expressed a need for some degree of help or support from formal or informal sources of support. Some only expressed a vague feeling of needing help but did not know what kind of help or where to get it, while others articulated emotional, practical, or health-related needs for help to manage the abusive experiences or prevent future victimization.

Some preferred emotional support given by informal helpers, e.g., friends and family, while others preferred formal helpers, e.g., health care professionals. Sometimes an informal helper acted as a bridge to obtaining formal help. Mixed feelings were often expressed about the experience of talking about abuse. It was found to be sensitive and difficult in the moment but generally positive in the long run. Some had experiences of talking to therapists who had given them practical assignments as parts of the therapy, e.g., painting or writing, which had been helpful. However, the most commonly expressed need was simply someone to talk to. One woman still living with an abusive intimate partner said:

*“I also have to get it off my chest sometime, otherwise I will break.” (Woman, aged 71)*

Practical support sought after could be financial aid, a new place to live, or a general realization of needing others when deciding to leave an abusive intimate partner relationship. For some, the need was related to physical dependence due to an aging body, e.g., not being able to live alone in an apartment or to care for a pet alone due to physical illness. For older adults with high level of dependence, it was suggested that a proxy would be valuable; someone who could help to speak up for the older adult’s rights, especially in relation to health and social care. A combination of different forms of help was often needed. Some expressed a desire for medical help to treat their traumatic experiences, e.g., counseling or medical help for sleep disturbances. In some cases, the informant stated that it was really the abuser who needed the help, including medical help, e.g., for alcohol dependence. However, it was acknowledged that this might be difficult, as such help would be dependent on the abuser’s willingness to accept help. One woman living with an abusive husband talked about the alcohol being a reoccurring problem in their relationship and a reason for the violence. However, her husband was not willing to seek help for it:

*“I talked with him about the wine. I said, you absolutely can not drink any wine. I have asked him the same thing before, but no, he falls back into it you know. I think it will be the same thing again. His father was an alcoholic […] And then they [staff at the psychiatric clinic where she had sought help] said that it is not you who should come, it is your husband. I could not get him to go [seek help]. He does not want anything do with such things. No, he does not” (Woman, aged 71)*

Another form of help needed was someone who could prevent the abuse in the situation, e.g., bystanders who would react and intervene. One man had been subjected to neglect as well as psychological and physical abuse by health care professionals while hospitalized. On one instance, two nurses had been in the room and one of them had struck him on his head with a pillow.

*“The [other] nurse who was present at night, when it happened, she said: ‘That’s enough now.’ […] She could have done more. She could have reported it to the manager on the ward.” (Man, aged 76)*

In some cases, professionals had intervened to end the abuse. One older woman had told her general practitioner about sexual harassment she had endured from a physician at a different clinic. The general practitioner had immediately taken charge of the situation, making sure that the abuse had ended, and that the abuser was reported.

For some informants, ageist attitudes among care professionals were part of the abusive experiences. In such cases, it was sometimes suggested that professionals need to be better at considering older adults not as a homogeneous group but rather as individuals with different experiences and needs, which might be a preventive effort:

*”…than maybe that person [the home care staff] is a bit patronizing and do not consider that one has been a professional… and only [see] an older person, that is the common thing. […] …within home care, it is usually so, that they tar everyone with the same brush, one is an old-timer. […] [Interviewer: What could be done to…?] Educating the staff of course. To understand that the older person has a background in whatever it is. And that, for example, the home care staff know of that [background] […], perhaps educating [the staff] to understand the individual.” (Woman, aged 84)*

### Internal resistance toward disclosing

Even though many informants had a strong need to talk about their abusive experiences and seek help, it was evident from the interviews that many struggled with an inner resistance against doing so. They expressed strong feelings of shame or that they considered their experiences to be too private to share with others. For those living with abusive partners, fear of reprisals was also articulated:

*“Also I am afraid. Because he said, well if you talk to others – then hell! He yelled at me. So no, I do not dare. I am afraid something will happen to me.” (Woman, aged 71)*

The informants also underlined that the decision to disclose experiences of abuse cannot be forced; the older adult must be ready to talk about the abuse, and should not be pressured into doing so. Likewise, it is important that professionals respect the victim’s wishes, e.g., a decision not to pursue a police report. Even though the informants expressed positive attitudes toward health care professionals who showed an interest in this issue and asked questions about abuse, several informants said that they were not sure they would disclose even if they had been asked questions. One woman who had a history of intimate partner violence as well as childhood abuse and elder abuse in health care talked about being asked questions in health care:

*“Yes, I would have liked it [to be asked questions about abuse]. But the question remains whether I would have been brave enough to tell. But I would have liked to be asked the question, because then… I would feel more validated then: ‘Someone suspects that something is very wrong here.’” (Woman, aged 67)*

Factors related to aging were also relevant for the informants. Some expressed resignation about their situation. They had lived with their abuse for so long that they seemed to have given up on getting out of it. This feeling of resignation could relate to both leaving an abusive relationship and seeking help to manage the abuse. For some, physical dependence on others kept them from seeking help. One woman explained that if she were to seek help, her abusive husband would have to be alongside her wheelchair and see where she went. This made it feel impossible for her to make contact with formal caregivers. Even when respondents were not as explicitly dependent on the abuser, physical illness kept them from seeking help. Several expressed that they did not have the energy to make life changes due to their illness. One woman explained that leaving her abusive partner would probably entail moving to a nursing home as well as having to get rid of two beloved dogs:

*“Well, it’s the dogs of course, I have two… and I would like to bring them with me […] But he thinks that I cannot handle it. I cannot manage to have my own apartment or anything – so that is, that is difficult […]. I have thought about doing something about it […] But I do not have the energy now you know, no it will have to remain as it is […] So it is really I who should react, but I do not have the energy now, due to my heart [condition].” (Woman, aged 71)*

Consideration for others also created resistance to disclosure and complicated the help-seeking process. Sometimes it was an ambivalent relationship with the abuser that led to resistance, but more often informants articulated strong concerns about how disclosure would affect other people they cared for, e.g., family members. One woman feared that talking about the abuse her brother-in-law had put her through would hurt her sister deeply, so she kept it a secret. Likewise, she did not want her children to know about the abuse her husband, their father, had put her through.

*“But because they really cared for their father and because he has never done anything to them… One cannot take that from them. I can’t do that. If you consider that I have so many illnesses now, so I don’t have much time left […] Perhaps it is only a short time and then I get to rest. So, the children should have their perception of the situation, at least they have never been harmed [by this].” (Woman, aged 66)*

Although several informants described the importance of friendships in which they could find comfort, some instead emphasized that you should not burden your friends with all your misery. Some also took a similar stand in relation to health care providers:

*“Anyway, [she is] a really good physician […] But I don’t want to burden her with this.” (Man, aged 88)*

### External facilitators or barriers for disclosure

When deciding whether to disclose abusive experiences to health care professionals, previous experiences and expectations regarding the health care system were important. Low expectations and trust in support systems were recurrently expressed. Some informants expected – sometimes due to previous experiences – that they would not be believed by health care professionals, that the person they told would say that they only had themselves to blame, or that they would not receive adequate help. Low faith in the patient–health care provider confidentiality was also expressed by some as the reason they did not seek professional help, e.g., in the words of one woman who had a history of childhood abuse and was currently living with an abusive intimate partner:

*”I don’t have confidence in them [staff at the primary health care centre]. [Name of town] is a small community with a lot of mouths. [Patient-provider] confidentiality don’t really apply there, as it should. So, I would never ever go [to the primary health care centre for help]” (Woman, aged 66)*

However, health care professionals who had shown interest and commitment were repeatedly recognized for their importance. The significance of trust built over time and a good relationship with a health care provider before disclosing abuse was articulated in several interviews. One man subjected to childhood abuse, as well as elder abuse by a sister and a health care professional had started to share his story with a counselor at the clinic where he was currently treated:

*“I have an illness and have been admitted several times and have regular check-ups too, once a week…. [With time] I have gained trust in some [of the staff].” (Man, aged 67)*

Health care professionals asking questions about abuse was generally appreciated by the informants. When the older adult felt that a health care professional was really interested in their history, this facilitated disclosure. Although an existing trusting relationship was repeatedly recognized as important, some informants described that they had decided to share their story without prior knowledge of the health care provider. The need for a prior alliance seemed to be related to the degree of urgency for sharing their experiences. When asked about the idea of using a written screening form to identify patients with abusive experiences in health care (like the one used in this study), several informants reported that they thought that was a good idea and that such a procedure could potentially facilitate disclosing experiences of abuse, e.g., one woman who had a history of intimate partner violence as well as childhood abuse and had been subjected to elder abuse in health care said:

*“It would have made it somewhat easier [with a questionnaire], to tell someone about what had happened. Sometimes it is easier to fill out a questionnaire…” (Woman, aged 67)*

Some informants had been dependent on the watchfulness of others for their help-seeking, e.g., someone in the older adult’s vicinity had noticed their predicament and helped them to seek help. Also, help-seeking was sometimes triggered by life circumstances, e.g., the death of a parent or watching a television program about abuse.

*“… No I think, during my career, I swept it away. Then they [the bad memories] start to resurface when you have more time to think about it, when something happens. Then you realize that it is like unresolved traumas, really.” (Woman, aged 69)*

Societal changes also had an impact on the help-seeking process. The international “#MeToo” social movement that challenged the silence surrounding sexual abuse was a recurrent theme during the interviews. Several informants had started thinking and talking about their experiences because of the movement’s influence on the public debate.

### The complexity of factors influencing how abusive experiences are managed

The different themes identified in this study were connected to and affected each other. The complexity of managing abusive experiences can be illustrated by one participant’s comprehensive story: Her stepfather had sexually, physically, and emotionally abused her during childhood, and she still suffered extensively as a result. For a long time, she had used avoidant coping, trying not to think about the abuse, and had told no one. Still, the memories and feelings had always been there in the background, affecting her well-being. During the fall of 2017, when #MeToo strongly influenced the public debate in Sweden, everything resurfaced for her, and she started feeling depressed. She was in regular contact with a nurse she trusted at the primary health care center due to a chronic illness. The nurse noticed that something was wrong, and because the nurse showed commitment and interest, the old woman decided to disclose her experiences and her suffering. The nurse then helped her to get a doctor’s appointment and the woman was diagnosed with post-traumatic stress disorder. At the time of the interview, she was waiting to start treatment sessions with a therapist. However, the help-seeking had been delayed by aging and illness, as the psychotherapy had to be postponed due to the same physical illness that had also led to her current hospitalization.

## Discussion

We identified three themes pertaining to strategies used by older adults to manage abusive experiences: self-reliant coping, restoring dignity in relation to others, and needing help from formal or informal sources of support. Strategies used to manage abuse that involve others, including help-seeking, are naturally reliant on disclosing experiences of abuse to e.g., friends, family, and professionals. Hence, the other two themes identified in this study (inner resistance toward disclosing and external barriers and facilitators for disclosure) are also central components of managing abuse.

### Managing the abusive experiences

The informants often used self-reliant and disengagement coping strategies to manage the abuse, e.g., avoidance and minimizing the experience. These were often conveyed as unsuccessful strategies in the long run, and several informants elaborated on how other life circumstances had triggered remembrance of the abuse and suffering long after it had occurred. In accordance with this, disengagement coping strategies are usually considered to be less effective at reducing distress over time than engagement coping strategies [21, 24]. Another coping strategy used by several informants was focusing on positive things in their lives, similar to behavioral activation. Likewise, victims have been reported to focus on hobbies or work to manage their experiences of elder abuse [34]. Redirecting attention or shifting the perspective from stressors to positive actions or emotions have previously been described as adaptive coping strategies [24].

Some informants had increased safety behaviors in response to the abuse, e.g., hiding jewelry or writing down the names of the home care staff who came to help after experiencing financial abuse. Similarly, changing behaviors have previously been reported as a reaction to elder abuse [34]. We interpreted such behaviors as a form of active self-reliant coping, i.e., informants tried to control potentially re-abusing circumstances and thereby minimize the risk of future victimization. The effectiveness of such measures remains unclear, but the examples illustrate the vulnerability of older adults with functional dependencies. They often have no choice but to continue to welcome staff in their home or remain on the hospital ward even after abuse occurred, most often with no control over who comes to help them. This vulnerability is probably part of the explanation why several informants described it as particularly difficult to stand up and defend oneself when abuse occurred within health care.

Some informants attributed the blame for abuse they endured to individual care professionals, but it was also common to blame the care organization and its limited financial resources for experiences of abuse, especially neglect. Although societal resources are often lacking, blaming the system may also be interpreted as a coping strategy. When dependent on others for help, it is likely to be easier to manage the situation if the helpers are not viewed as perpetrators of abuse. Similarly, previous research found that functionally impaired older adults reported emotional abuse to be less severe if they were also dependent on the perpetrator for caregiving [40]. In that study, the abuse was perceived as less severe when it could be attributed to some extrinsic factor, e.g., stress. Seemingly as an expression of family loyalty, the understanding of caregiver burden was found to be limited to relatives, i.e., when the perpetrator was a close family member the abuse was interpreted as less serious than when the abuser was a paid caregiver [40]. By contrast, several informants in this study also reported an understanding of caregiver burden for professionals or blamed the organization, rather than the individual.

Although self-reliant coping strategies were common, we also found many examples of managing abusive experiences with the help of others, e.g., reacting to the abuse by standing up for oneself or actively seeking emotional support from friends, family, and professionals. Previous research found that abused older women who used such active coping strategies reported a higher quality of life than those who used passive coping strategies, and that active coping is especially beneficial for older adults who are subjected to the most severe forms of abuse [23]. This coincides with our finding that an important part of restoring dignity in relation to others was to stand up for oneself and to speak out against the abuser. However, such behaviors were more clearly articulated among those experiencing abuse for the first time as older adults than those reporting repeat victimization, i.e., polyvictims. Although it needs to be investigated quantitatively whether this is a general pattern, this finding supports previous claims that a life-course perspective is important in research on elder abuse [16–20]. Future studies should investigate not only how previous victimization affects late life exposure to abuse and its health consequences, but also how previous victimization affects how older adults manage abusive experiences.

Needing help from formal or informal sources of support was repeatedly articulated by respondents. There is a shortage of research into evidence-based best practice to care for victims of elder abuse [11, 33, 41, 42]. The needs of abused older adults are often complex, not least considering that the perpetrator of abuse may be the primary provider of both practical help and social support. An example of this complexity was when the benefits of leaving an abusive intimate partner relationship had to be weighed up against having to move into assisted living and losing a beloved pet that could not be cared for without the partner’s assistance. Such complexity must be considered when providing help. However, the most commonly sought form of help by the informants in this study was rather simplistic: “Someone to talk to.” The older adults had different preferences regarding seeking emotional support from formal or informal helpers, but the fundamental need to be validated was important for encounters both with professionals and in relation to friends and family. Likewise, being treated respectfully and being listened to was important for the process of disclosure. This coincides well with trauma-informed care, previously recommended when caring for older adults who are subjected to abuse [33]. It is also consistent with expectations on health care providers expressed by female victims of intimate partner violence [43–45].

A need for practical and medical help was also articulated. Previous research found that most victims of elder abuse needed more help than they currently received [28]. Some of the respondents in this study had problems specifying the kind of help they wanted, or did not know where to find it. A lack of knowledge about support systems, difficulties navigating services, or not believing that support systems have the potential to help have been previously reported as barriers for help-seeking [12, 28, 29, 31]. One suggestion made by some informants was the introduction of a proxy who could act in the interests of older adults. Sweden has a fiduciary system in place, as well aid assessors who should ensure that older adults’ needs are provided for. However, it seems that this is not always enough, and some informants felt that they needed someone to fight for their rights to welfare. Previously, case coordination has been reported as a need from survivors, and this coincides with our findings and previous research showing that many victims do not know where to seek help or how to navigate help they know is available [28].

Several informants in this study underlined that professionals should not pressure them into disclosing, and that it is important to respect the older adult’s wishes, e.g., not wanting to pursue a police report. This is in line with previous research findings, whereby professionals need to be aware that ideas about what constitutes case resolution may vary between victims as well as between victims and professionals [25, 29, 32]. Also providing help to the abuser was suggested by some of the informants, and such a need has been emphasized in previous research [29]. Giving control and choices to the victim while prioritizing their need for safety and respect are important parts of trauma-informed care, and this has been suggested as an important approach for providing the best possible care for victims of violence, including elder abuse [18, 33].

### Help-seeking: Barriers and facilitators for disclosure

A number of barriers and facilitators for disclosure were identified in this study, and several of them can be recognized from previous studies. Shame is a well-known feeling after abuse and a barrier for help-seeking reported in both the elder abuse literature and in the related field of intimate partner violence[28, 29, 32, 34, 35, 46] that we also found to be important in this study. Even when the abuse had ended a long time ago, shame was still a factor to consider and often the reason why the abuse had been kept a secret over the years.

Also confirming previous research, we found that a strong barrier for help-seeking was consideration of others, mainly the perpetrator or other family members [12, 29, 30, 34, 35]. The victim needed to feel sure that the intervention to help them does not harm themselves or their family, often including the abuser. Otherwise, they are likely to refrain from seeking help [29, 32]. In a study of Chinese older adults, similar findings were interpreted as an expression of cultural beliefs that family members should sacrifice themselves for the greater good of the family [12]. Sweden is a rather individualistic and secular society where such cultural expectations are not prominent [47]. However, we found that consideration of family members was a barrier toward help-seeking, and protecting adult children’s feelings was a motivator for keeping the abuse a secret. The physical limitations of the aging body were also important. As in previous studies, we found that care dependence was a barrier to disclosure [26, 29, 31, 48]. Also, on a more fundamental level, access to help could be restricted due to physical limitations, e.g., difficulties with transportation for wheelchair users.

Engaging and caring professionals were a facilitator for help-seeking. Likewise, previous studies, both about elder abuse and intimate partner violence, have report the importance of positive relations for disclosure in health care [25, 28, 29, 35, 43–45] as well as for retainment in elder abuse response programs [25]. When trust had been built in a long-standing care relationship, e.g., when treating a chronic disease, this facilitated disclosure. A high perceived need for help also facilitated disclosure, and the informants underlined the importance of feeling ready to talk about their experiences. However, help-seeking could also be triggered by societal or personal changes in life, sometimes seemingly unrelated to the experiences of abuse. Similar findings were previously reported in a Swedish study of male victims of violence [49].

Sweden does not have a mandatory report system for elder abuse. It is difficult to estimate the effect such a system would have on the process to disclose abuse and seek help. On the one hand, considering the difficulties expressed by informants concerning navigating support system and how feeling of shame made it more difficult to disclose abuse, it might be beneficial to introduce such as system to make sure more victims are given appropriate help. On the other hand, informants in this study also expressed the importance of feeling ready to disclose and that it is important to respect the older adults’ whishes when providing help. Both may be threatened by mandatory reporting, which hence may impede older adults’ trust in caregivers and thereby alienate some victims and keep them from seeking help.

### Clinical implications

The importance of others for managing experiences of abuse was evident in our findings, but formal sources of support were reported as being difficult to navigate. The helper’s ability to build trust was also a strong facilitator for disclosure, but several informants had experienced poor encounters with professionals. The same pattern has been reported in studies concerning health care response to intimate partner violence [44, 45, 50]. Altogether, this underlines the need to improve the societal response to victims of abuse in general and older adults in particular.. Training for professionals could potentially help increase their understanding of the issue and help more professionals develop the skills to build trust. Access to help that is adapted to the specific needs and preferences of the individual is also needed. An important task would be developing more easily navigated response systems that can respond to the complex needs of older adults. As previously suggested, multi-professional teams may be one example of a way forward [25, 51].

Elder abuse sometimes occurs in health or social care. How to prevent this was a recurrent theme in the interviews. Repeated suggestions from participants included more allocated resources and education for staff. The informants also wanted the care system to learn from their individual experiences so that future victimizations could be prevented.

### Limitations

Research findings are always affected to some extent by the researchers’ previous experiences and interpretations. We managed the risk of reproducing preconceived ideas in several ways. First, we used field notes to make our own preconceived ideas visible and to stimulate reflexivity. Second, although only the first author coded all interviews, the last author coded six interviews to ensure similar interpretations. The last author had previously coded all interviews describing the experiences of elder abuse for a separate study [20], and was hence well acquainted with the material. Results were continuously discussed between the first and last authors. This triangulation of coding and interpretation of findings aimed to strengthen the credibility of the study through reflexivity by contesting and supplementing each other’s understanding of the material [52]. To further facilitate the readers’ judgement of trustworthiness, we included Fig. 1 as well as quotations from the interviews.

The recruitment method likely affected the results and more specifically the transferability of results. Contrary to some previous studies about help-seeking for elder abuse, we recruited participants consecutively from patients treated at a hospital, i.e., not from victim services or through advertisements. This sampling method likely contributed to our diverse sample including both victims in great suffering and need of help and those with less prominent needs, which strengthens the credibility of our findings. By including informants with very diverse abusive experiences in the same study it was possible to look for general patterns in how older adults manage experiences of abuse. However, it is likely that both type of abusive experience and relationship with the abuser affect how the victims manage the situations and it would be valuable in future studies to focus on specific types of abuse to better inform interventions in e.g., health care. At the same time, it should be noted that poly-victimization was found to be common and that both multiple experiences of abuse and previous encounters with formal and informal helpers affected how the older adult managed their experiences. Hence, there is a risk that a narrow focus on specific types of abuse miss important conjunctions between different types of victimization. It is a limitation of this study that none of the informants reported life-threatening forms of abuse and hence our results may not be transferrable to the group of older adults who endure the most severe forms of abuse. Also, it should be noted that only three informants (10%) lived in assisted living facilities. Considering that ill-health and functional dependence were found to be important internal and external barriers for disclosure, future studies should focus specifically on how older adults living at such facilities manage experiences of abuse.

## Conclusions

This is one of few studies investigating how older adults manage experiences of abuse. We found that they use a variety of coping strategies; some are self-reliant, but older adults often manage their abusive experiences with the help of others. For some, disclosure is a difficult process that is hampered by both internal and external barriers. Therefore, efforts need to be directed at facilitating the process of disclosure, and by extension help-seeking. Such efforts could include training health and social care professionals on issues related to elder abuse. Easily navigated support systems that can respond to the complex needs of older adults also need to be developed.

## Supplementary Information

**Additional file 1.** INTERVIEW GUIDE (for two parallel studies).

## Data Availability

The datasets generated and analyzed during the current study are not publicly available, and are not available from the corresponding author on request due to participant privacy and confidentiality.
