# Supplementary material for: Managing abusive experiences: a qualitative study among older adults in Sweden
Source: BMC Geriatr. 2022 May 26;22:456. doi: 10.1186/s12877-022-03143-y (PMC9137123; doi:10.1186/s12877-022-03143-y)
Supplement: Supplementary file 1 — Additional file 1. INTERVIEW GUIDE (for two parallel studies). [file 12877_2022_3143_MOESM1_ESM.pdf]

Additional file 1. INTERVIEW GUIDE (for two parallel studies)

AIM I: Explore the victims' experiences of elder abuse and how it affected them *[Mainly corresponding to another study about experiences of abuse, but also to how victims had managed their abusive experiences]*

1. Can you tell me a little more about what you've been through?
   Probing: what, who, when, duration?

2a. What thoughts do you have today when you think about it?
2b. What feelings do you have today when you think about it?

3. In what ways did your experiences affect you?
   Probing: Affected everyday life? Affected your behavior? Now, before?

AIM II Explore preferred intervention for elder abuse among victims *[Corresponding to this study]*

1. Have you ever sought help because of your experiences [of abuse]?
   Probing: Where? When? What kind of help did you receive? How did it feel?
   If no help was sought: Have you ever felt the need to get help to manage your experiences? When the abuse occurred? Afterwards? Now?
2. Do you think you got the help you need?
   Probing: What did you find to be beneficial? Less beneficial?
3. What kind of help did you want? Now and back then [when the abuse occured]? What would be the main goal with receiving help? Now and back then [when the abuse occured]?
   Probing: Practical help? Economical? Help to leave the abuser? Help to manage the situation or relationship? Compare [the help needed] now and then?
4. How do you think that help is best given?
   Probing: How shoud it be organised? Who should give the help? How should victims not that it is available?
5. Do you think family or friends should be involved in the help given? In what way?
6. What advice would you like to give to health care professionals working with older adults subjected to abuse?

FINAL QUESTIONS, corresponding to both aims above:

1. Is there anything else you would like to add? Is there anything that we've forgotten to talk about?

After the interview:

Check results of depression assessment, consider suicide risk or need for referral.
